# A Lesson in Diagnosis

**Published:** 1872-12

**Authors:** 


### A Lesson on Diagnosis.

Dr. Waters, in a clinical lecture reported in the London *Lancet*, gives the following good advice to the young practioner:

"It may seem to you an easy matter to make out the nature of a case. A man of experience will put a few leading questions, make perhaps only a slight physical examination, and at once pronounce a correct opinion of the existing ailment; and you may imagine that you will readily be able to do the same. But do not be mistaken. This power of rapid diagnosis has been the result of long observation and great painstaking; and you will find even that the man of the most matured judgment will often spend a long time over a case—will examine carefully all its details—before he will venture on an opinion of its nature. If you wish to attain to the power of rapid diagnosis in ordinary cases—you must begin by examining with the greatest care every detail of a series of cases which are the best marked instances of their kind. The study of these will prepare you to understand the varieties and complicacations which so constantly present themselves at the bedside."—*Medical Herald.*

